# Synergistic Rifabutin and Colistin Reduce Emergence of Resistance When Treating Acinetobacter baumannii

**DOI:** 10.1128/AAC.02204-20

**Published:** 2021-03-18

**Authors:** Jiaqi Cheng, Jun Yan, Zeferino Reyna, Matt Slarve, Peggy Lu, Brad Spellberg, Brian Luna

**Affiliations:** a Department of Molecular Microbiology and Immunology, Keck School of Medicine at USC, Los Angeles, California, USA; b Los Angeles County-USC (LAC+USC) Medical Center, Los Angeles, California, USA

**Keywords:** *Acinetobacter baumannii*, antibiotic resistance, colistin, rifabutin

## Abstract

Recently, we reported rifabutin hyperactivity against Acinetobacter baumannii. We sought to characterize potential interactions between rifabutin and colistin, the last-resort drug for carbapenem-resistant infections. Rifabutin and colistin were synergistic *in vitro* and *in vivo*, and low-dose colistin significantly suppressed emergence of resistance to rifabutin. Thus, this combination is a promising therapeutic option for highly resistant A. baumannii infections.

## INTRODUCTION

Carbapenem-resistant Acinetobacter baumannii poses a significant problem for health care and has been identified by the World Health Organization (WHO) and the Centers for Disease Control (CDC) as a priority pathogen for which new antibiotics are needed (1–3). We recently conducted a modified compound screen assay using RPMI broth with serum, a medium that better models the *in vivo* physiologic environment than rich media (e.g., Mueller-Hinton II broth). We found that rifabutin (RBT) possesses previously unrecognized hyperactivity (MIC < 0.05 mg/liter) against A. baumannii but only in physiological media and not in rich media (4). Additionally, A. baumannii-infected mice that were treated with a 300-fold lower dose of RBT had significantly better survival than mice treated with rifampin (RIF) (4).

In a randomized controlled trial, adjunctive RIF significantly improved microbiological eradication when added to colistin (COL) for treatment of extremely drug-resistant A. baumannii infections, but it did not significantly improve mortality (5). Since RBT is much more potent than RIF *in vitro* and *in vivo*, we hypothesized that combination therapy with RBT and COL might be a more promising option to treat carbapenem-resistant A. baumannii infections. Here, we sought to determine the nature of any interaction between RBT and COL and define the impact of COL on emergence of resistance to RBT.

We began by determining monotherapy MICs. Clinical isolates of A. baumannii were tested for susceptibility toward RBT, RIF, and COL in Mueller-Hinton II Broth (MHII) and RPMI with serum. The 10 selected isolates comprised a panel with different susceptibilities against RBT and COL, including antibiotic-sensitive, single-antibiotic-resistant, and double-antibiotic-resistant (Table 1). Consistent with what we previously reported (4), there was no difference between the MICs of RBT and RIF in MHII medium. However, in RPMI with serum medium, the MICs of RBT were lower than those of RIF for RBT-hypersensitive (MIC < 0.05 mg/liter) isolates. Of great interest was that COL-resistant strains, as defined by MICs greater than 2 mg/liter in MHII medium, had significantly lower COL MICs in RPMI with serum, again suggesting that more physiologically relevant culture medium alters apparent susceptibility of antibiotics compared to rich broth *in vitro*.

**TABLE 1:** MICs of RBT, RIF, and COL alone and drug-drug interactions of RBT + COL or RIF + COL for A. baumannii^*a*^

		Single-drug MIC (mg/liter)/(synergy [S], no interaction [N], antagonism [A] with COL)		
Strain	Medium	COL	RBT	RIF
HUMC1	MHII	0.25	1.56 (S)	1.56 (N)
	RPMI + 10% serum	0.125	0.05 (N)	12.50 (N)
HUMC1 Δ*fhuE*	MHII	0.25	12.50 (S)	1.56 (S)
	RPMI + 10% serum	0.125	1.56 (N)	12.50 (N)
LAC-4	MHII	0.125	3.13 (S)	0.78 (S)
	RPMI + 10% serum	0.125	0.78 (N)	1.56 (N)
LAC-4 Col-R	MHII	64	3.13 (S)	0.78 (S)
	RPMI + 10% serum	0.125	0.78 (S)	3.13 (S)
VA-AB41	MHII	2	3.13 (S)	1.56 (S)
	RPMI + 10% serum	0.125	0.39 (S)	6.25 (S)
AB5075	MHII	0.25	1.56 (S)	1.56 (S)
	RPMI + 10% serum	0.25	0.05 (S)	>25.00 (S)
AB5075 tn::fhuE	MHII	0.5	3.13 (S)	1.56 (S)
	RPMI + 10% serum	0.25	0.39 (N)	12.50 (N)
C8	MHII	>64	12.50 (S)	12.50 (S)
	RPMI + 10% serum	0.125	0.05 (S)	6.25 (S)
C14	MHII	32	>25.00 (S)	6.25 (S)
	RPMI + 10% serum	0.5	>25.00 (S)	25.00 (S)
AR0299	MHII	1	>25.00 (S)	>25.00 (S)
	RPMI + 10% serum	1	>25.00 (S)	>25.00 (S)

a Drug-drug interactions were evaluated by calculating the fractional inhibitory concentration index (FICI). Synergy was defined by FICI ≤0.5, no interaction by FICI >0.5 to ≤4.0, and antagonism by FICI >4.0.

Next, RIF and RBT MICs were determined in combination with COL. For most strains tested, we found that both RIF and RBT were synergistic (fractional inhibition concentration < 0.5) with COL *in vitro* (Table 1). However, for some strains, synergy was not observed in both media. The drugs did not demonstrate antagonism against any strain in either medium.

**FIG 1:**
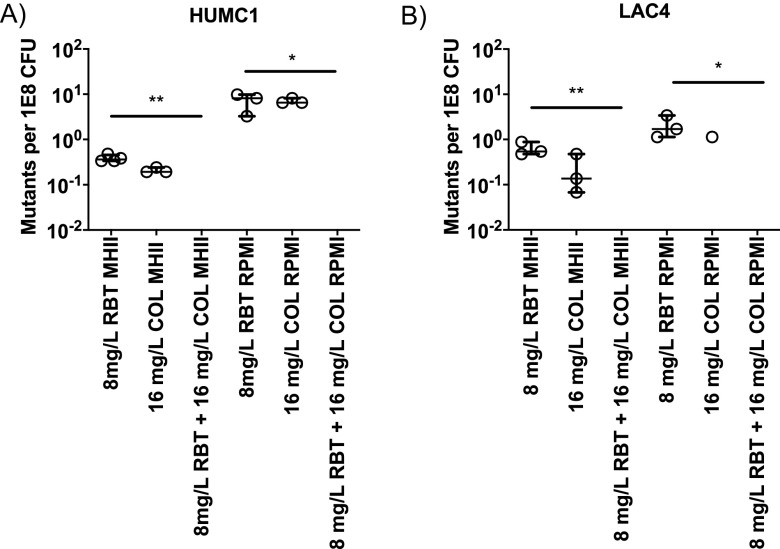
Selection of antibiotic-resistant mutants by high inoculum plating. A. baumannii clinical isolates were cultured in MHII or RPMI medium overnight, and mutants were selected by plating bacteria on MHII or RPMI drug plates containing 8 mg/liter of RBT, 16 mg/liter of COL, or the combination of both antibiotics. No CFU were observed in any of the combination treatment groups. (A) For HUMC1, there was a significant difference between the combination group and RBT alone in MHII (Kruskal-Wallis, *P* = 0.008) and RPMI (Kruskal-Wallis, *P* = 0.027). (B) For LAC-4, there was a significant difference between the combination group and RBT alone in MHII (Kruskal-Wallis, *P* = 0.008) and RPMI (Kruskal-Wallis, *P* = 0.017). *n* = 3 for all groups. The median and interquartile range were plotted for all graphs.

One of the concerns with using rifamycins as monotherapy is that bacteria may rapidly acquire resistance. We sought to determine whether the addition of COL could suppress the emergence of RBT resistance in A. baumannii (Fig. 1). By plating high bacterial inocula, we found that the combination of RBT and COL significantly reduced the emergence of resistance to RBT (Kruskal-Wallis, *P* < 0.05) (Fig. 1).

**FIG 2:**
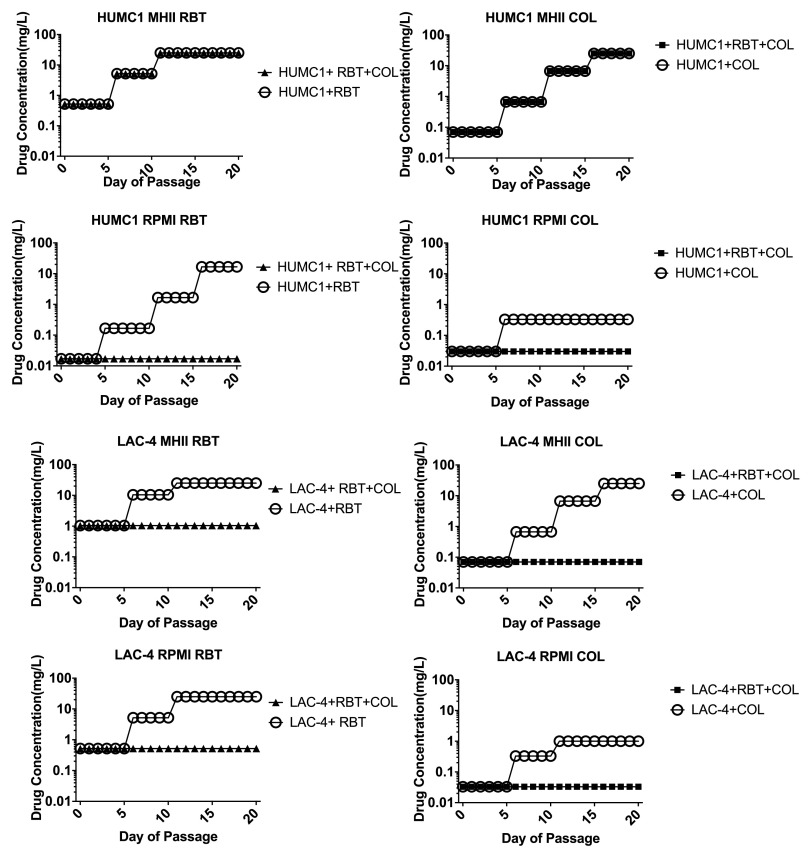
Selection of antibiotic-resistant mutant by sub-MIC over a 20-day serial passage. A. baumannii clinical isolates were cultured in MHII or RPMI medium with one-third the MIC of RBT, COL, or both antibiotics. After each 5-day passage, the resistance of bacteria was determined by plating the bacteria on 3× and 10× the current passage antibiotic concentration. The starting concentration for the next 5 days was increased based on the plating result. For all groups cultured in RPMI, the combination treatment suppressed the emergence of resistance compared to monotherapy groups.

To allow for the accumulation of low-resistance-conferring mutations, we also conducted low inoculum serial passage of bacteria by serially passaging 20 times in sub-MIC conditions in MHII and RPMI without serum (Fig. 2). Antibiotic susceptibility was tested every 5 days and the antibiotic concentration used for culture was increased if possible. As expected, culturing bacteria with subinhibitory concentrations of single antibiotics fostered emergence of resistance. However, the combination of RBT and COL suppressed emergence of resistance to RBT for most strains in both media conditions (with the exception being HUMC1 in MHII medium).

**FIG 3:**
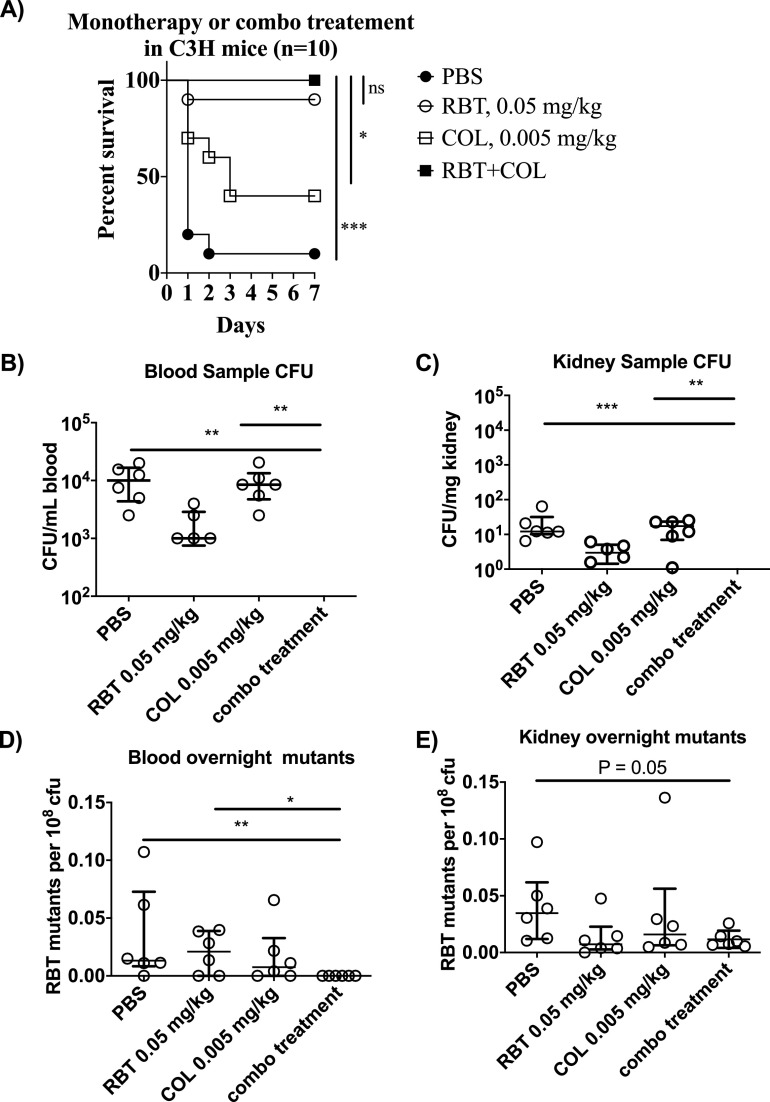
Efficacy of rifabutin colistin combination treatment *in vivo*. (A) C3HeB/FeJ mice (*n* = 10 per group) were infected with 1.2 × 10^7^ to 3.9 × 10^7^ CFU of the hypervirulent (100% lethal dose [LD_100_] < 2 × 10^7^ CFU) carbapenem-resistant A. baumannii HUMC1 (9, 10) and treated with PBS, 0.05 mg/kg RBT, 0.005 mg/kg COL, or a combination of RBT and COL. There was a significant difference comparing the RBT+COL group to the PBS (<0.001, log rank) and COL monotherapy groups (0.0113, log rank). There was no significant difference between the combination treatment group and the RBT monotherapy group. (B) C3HeB/FeJ mice (*n* = 6 per group) were infected with 5 × 10^7^ CFU of A. baumannii HUMC1. Mice were treated once after infection with PBS, 0.05 mg/kg RBT (subtherapeutic), 0.005 mg/kg COL (subtherapeutic), or RBT + COL. Blood and kidney samples were collected 24 h postinfection and kidneys were weighed and homogenized. Blood (B) and kidney (C) homogenates were enumerated on TSA plates and results were recorded. No CFU were observed for the RBT + COL treatment group in the blood and kidney homogenate. In the blood, there was a significant difference between RBT + COL combination compared to PBS (Kruskal-Wallis, *P* = 0.001) and COL (Kruskal-Wallis, *P* = 0.001). In the kidneys, there was a significant difference between RBT + COL combination compared to PBS (Kruskal-Wallis, *P* = 0.0006) and COL (Kruskal-Wallis, *P* = 0.001). (D) 100 μl blood and (E) 100 μl kidney homogenates were used to inoculate 10 ml of TSB, the outgrowths from the overnight cultures were serially diluted and plated on drug and nondrug TSA plates, and the frequencies of resistant mutants were enumerated. In the outgrowth from the blood sample, there was a significant difference between RBT + COL combination compared to PBS (Kruskal-Wallis, *P* = 0.008) and RBT (Kruskal-Wallis, *P* = 0.026). In the outgrowth from the kidney sample, there was a notable but not significant difference between RBT + COL and PBS (Kruskal-Wallis, *P* = 0.05). The median and interquartile range were plotted for all graphs.

Next, we evaluated the effect of combination therapy *in vivo*. As suggested by prior results (4), we confirmed that administration of combination RBT + COL therapy to A. baumannii-infected mice resulted in superior survival (Fig. 3A). We repeated the experiment to evaluate changes in bacterial density, which were not evaluated previously (Fig. 3B and C). At 24 h after infection, administration of combination therapy resulted in below-detectable-level bacterial density in blood and kidneys, while monotherapy groups had significantly higher bacterial density than the combination therapy group. Specifically, RBT + COL combination significantly reduced CFU in the blood and kidneys compared to phosphate-buffered saline (PBS) (Kruskal-Wallis, *P* ≤ 0.001 for all comparisons, Fig. 3B and C).

Bacterial density was too low to enable selection for RBT-resistant mutants on selective plates directly. Therefore, blood and kidney homogenates were used to seed overnight cultures, and the outgrowth was plated on Trypticase soy agar (TSA) plates supplemented with 8 mg/liter RBT (Fig. 3D and E). In the blood outgrowth culture, there were significantly fewer emergent mutants from organs taken from mice treated with RBT + COL than from those taken from mice treated with the PBS control (Kruskal-Wallis, *P* = 0.008) and with RBT monotherapy (Kruskal-Wallis, *P* = 0.026) (Fig. 3D). There was a notable, but not significant, difference between the RBT + COL combination and the PBS control group (Kruskal-Wallis, *P* = 0.05) (Fig. 3E).

We and others have recently described that RBT is hyperactive against A. baumannii because RBT, and not RIF, is able to rapidly traffic through the bacterial FhuE protein (4, 6). Furthermore, it has been postulated that COL can potentiate rifamycins by disrupting the membrane permeability of the bacteria and thus allow for increased intracellular trafficking of the rifamycin antibiotic (5, 7, 8). Thus, one potential explanation for diminished synergy in RPMI medium is that the bacterial cell is already highly permeable to RBT in RPMI due to the upregulation of FhuE proteins.

In summary, combination RBT + COL is a promising strategy to improve survival and reduce the emergence of resistance to RBT during treatment of A. baumannii infections. Importantly, a subtherapeutic dose of COL, which likely would result in diminished toxicity compared to that of standard dosing, was able to reduce the emergence of resistance to RBT during treatment of A. baumannii bacteremia in mice. These results indicate the promise of a low-dose COL + RBT combination regimen in the treatment of such infections.
